# A Commercially Available Portion-Controlled Diet Program Is More Effective for Weight Loss than a Self-Directed Diet: Results from a Randomized Clinical Trial

**DOI:** 10.3389/fnut.2017.00055

**Published:** 2017-11-07

**Authors:** Chad M. Cook, Courtney N. McCormick, Mandi Knowles, Valerie N. Kaden

**Affiliations:** ^1^Biofortis, Inc., Addison, IL, United States; ^2^Nutrisystem, Inc., Fort Washington, PA, United States

**Keywords:** overweight, obesity, fat mass, body circumference, pre-portioned foods

## Abstract

**Objective:**

To examine changes in weight and related outcomes in response to a commercial weight loss program compared to a self-directed diet in adults with overweight or obesity.

**Design:**

Participants were randomly assigned [stratified by body mass index (BMI) and age] to a commercial weight loss program (*n* = 38) or to a self-directed Dietary Approaches to Stop Hypertension (DASH) diet (*n* = 40) for a 16-week period. Daily energy intake goals were 1,500 kcal/d for men and 1,200 kcal/d for women, except for the first week of the commercial program (1,000 kcal/d). This study was registered at http://ClinicalTrials.gov (NCT03017443).

**Participants:**

Primarily Caucasian (71%) women (*n* = 61) and men (*n* = 17) from the greater metropolitan area of the city of Chicago, IL, USA. with a mean baseline BMI of 34.4 kg/m^2^, body weight of 95.7 kg, and age of 50.4 years.

**Results:**

Data = mean (95% CI). At week 16, the commercial program group lost significantly more body weight [−5.9 (−7.5, −4.3) kg vs. −1.8 (−2.9, −0.8) kg; or −6.4 vs. −1.8% of initial body weight, respectively], fat mass [−4.4 (−5.7, −3.1) kg vs. −1.2 (−2.1, −0.4) kg] and total body circumference (chest + waist + hip + upper arm + thigh) [−16.9 (−21.5, −12.3) cm vs. −5.8 (−9.0, −2.6) cm] (*p* < 0.01 for all). Additionally, more participants in the commercial program group lost a clinically meaningful amount of weight, defined as ≥5% of initial body weight, at week 16 (58% vs. 13%, *p* < 0.001).

**Conclusion:**

The commercial program resulted in greater weight loss and improvements in body composition/anthropometric parameters compared to a self-directed DASH diet over a 16-week period. Some important limitations were that no objective measurements of dietary intake or physical activity were collected to potentially ascertain the independent or combined effects of these components on weight loss (or lack thereof). Additionally, future research is warranted in order to understand the effects of this program, and similar programs, on longer term changes in body weight, in particular weight loss maintenance, as weight regain is common following the cessation of a structured weight loss intervention.

## Introduction

Factors contributing to weight gain are complex and obesity remains a multifaceted public health problem (1, 2). A reduction of ≥5% of initial body weight is often recommended as a primary step in managing or preventing comorbidities associated with obesity; however, recent advances in the understanding of the etiology of obesity, along with development of novel pharmaceutical and surgical treatment options, have yet to markedly impact obesity prevalence in the US population. Improving diet and physical activity remain the foundation of most obesity interventions.

In this context, the role of commercially available weight loss programs has received increased attention by the scientific community (3, 4). One approach shown to be useful in supporting weight loss is provision of pre-portioned foods and beverages (i.e., meal replacements or portion and calorie controlled foods) (5–8), especially compared to more conventional dietary advice, such as self-selected diets based on general concepts such as variety and moderation (9). Some commercially available weight loss programs utilize this approach, although data from randomized clinical trials documenting the degree of weight loss achievable with these programs are needed.

The primary objective of this study was to determine changes in body weight achievable in apparently healthy men and women with overweight or obesity following a commercial weight loss program, consisting of a nutritionally balanced calorie restricted diet *via* provision of portion-controlled foods and shakes, along with phone support from trained weight loss counselors, compared to a self-directed diet over a 16-week period. Key secondary objectives included determining changes in body composition, body circumference measures, and self-reported health-related quality of life, including sleep.

## Materials and Methods

### Study Design

This was a randomized, parallel group study conducted from April 2015 (first participant screened) to October 2015 (last participant completed). The intervention period was 16 weeks in duration, with clinic visits at baseline (week 0) and weeks 1, 2, 3, 4, 8, 12, and 16. Participants from the greater metropolitan area of the city of Chicago, IL, USA, that initially qualified *via* telephone screening and subsequently met all entry criteria at the screening visit (week 1) were randomized to one of the study arms. A statistician who was blinded to intervention assignment during data analysis generated a randomization list for intervention sequence using SAS PROC PLAN with a 1:1 allocation across groups, stratified by two body mass index (BMI) categories (25.00–29.99; 30.00–44.99 kg/m^2^) and three age categories (18–35; 36–54; 55–70 years). Numbered, opaque, and sealed envelopes concealing the allocation sequence were opened sequentially by a clinic staff member only after a participant was confirmed eligible for the study and the randomization number/test sequence was recorded with the subject’s source documentation. Blinding of study staff (with the exception of the statistician) or participants was not possible given the nature of the study interventions.

This study was conducted at Biofortis, Inc., Addison, IL, USA, in accordance with the recommendations of Good Clinical Practice Guidelines, the Declaration of Helsinki (10), and the United States 21 Code of Federal Regulations. The protocol was approved by an accredited Institutional Review Board (IntegReview IRB, Austin, TX, USA). All participants provided signed informed consent and authorization for disclosure of protected health information before any study specific procedures were carried out. At the time the study was initiated, the need to prospectively register as a clinical trial in a public database was not given proper consideration. Therefore, this study was registered *post hoc* at ClinicalTrials.gov at https://clinicaltrials.gov/ct2/show/NCT03017443?term=NCT03017443&rank=1 with the unique identifier NCT03017443.

### Study Participants

Men and non-pregnant, non-lactating women, 18–70 years old, each with a BMI 25.00–44.99 kg/m^2^ and in good general health on the basis of medical history and routine laboratory tests at screening were initially eligible for the study. Users of tobacco products were allowed, although these participants were required to maintain habitual use throughout the duration of the study. Premenopausal female participants were also required to have a regular menstrual cycle (ranging from 21 to 35 days) and were required to be willing to use a medically approved form of contraception throughout the study.

Participants who reported a weight change of ≥4.5 kg or used prescription medications for weight-reducing purposes within 6 months of screening, or who used weight loss supplements or other commercially available products/programs with the intent to lose weight within 1 month of screening, were excluded from the study. Participants with clinically significant abnormal routine laboratory test results or uncontrolled hypertension (resting systolic blood pressure ≥160 mmHg and/or a diastolic blood pressure ≥100 mm Hg) at screening were excluded. Additional exclusion criteria included the following: medical history indicating clinically relevant cardiac, renal, hepatic, endocrine (including diabetes mellitus at screening), pulmonary, biliary, gastrointestinal, pancreatic, or neurologic disorders; cancer in the past 2 years; known sensitivity to any of the ingredients in the study foods; a history of weight-reducing surgery; a history of eating disorders, extreme dietary habits, or alcohol abuse; use of thyroid hormones (except stable dose replacement therapy for at least 2 months prior to screening); or systemic corticosteroid use within 4 weeks of screening.

Participants were recruited *via* e-mail notifications to a database of individuals that had previously expressed interest in participating in research studies, *via* newspaper and social media/Internet-based advertisements, and by word of mouth.

### Dietary Interventions

#### Commercial Weight Loss Program

The commercial weight loss program utilized a two-phase approach. Participants followed a 1,000 kcal/day diet during the first week of the study, consisting of prepackaged portion-controlled foods and shakes, and were allowed to add non-starchy vegetables and no-calorie beverages within program guidelines during this first week. After the first week, daily energy intake targets were increased to 1,500 kcal/day (men) or 1,200 kcal/day (women) and participants were provided 7 breakfasts, 6 lunches, 6 dinners, and 7 (women) or 14 (men) snacks/desserts per week consisting of prepackaged portion-controlled foods. Participants were instructed to prepare one lunch and one dinner within program guidelines on their own each week. Prepackaged portion-controlled foods accounted for ~60% of daily energy intake, with recommended grocery food additions making up the balance. Written recommendations were provided to allow participants to self-select appropriate foods for these eating occasions that fit within program guidelines (~50% kcal/day from carbohydrate, ~25% kcal/day from protein, ~25% kcal/day from fat), and food intake was self-monitored through the use of a tracker (similar to a food checklist) consistent with the commercially available program. Participants also had access to support from weight loss counselors by phone at any time throughout the study period.

#### Self-Directed Diet

The control group was designed to mimic typical dietary advice that might be received in a primary care setting. Participants randomized to the self-directed diet received a limited intervention that included provision of publicly available information consistent with the Dietary Approaches to Stop Hypertension (DASH) diet (11), with instructions to utilize these dietary recommendations to consume a reduced calorie diet for weight loss. DASH is a well-balanced dietary pattern for the general public, encompassing fruits and vegetables, whole grains, legumes/nuts, and lean sources of protein such as fish and poultry, with limited consumption of added sugars, added fats, and red meat (12, 13). Daily energy intake targets were the same as the commercial weight loss program group (1,500 kcal/day for men and 1,200 kcal/day for women) with the exception of the first week of that program (1,000 kcal/day). To encourage study completion, participants randomized to the DASH group received vouchers to obtain 4 weeks of foods associated with the commercial weight loss program at the end of the study.

### Anthropometric and Blood Pressure Measurements

Body weight, body circumference (chest, waist, hip, upper arm, and thigh), and blood pressure measurements were obtained at baseline (week 0) and at weeks 1, 2, 3, 4, 8, 12, and 16 in the morning following an overnight fast (9–14 h). Height was obtained at screening without shoes to the nearest 0.1 cm using a wall mounted stadiometer (Seca model 2161814009, Hamburg, Germany). Body weight was measured using a digital floor scale (Health-O-Meter Professional model 349KLX, Boca Raton, FL) with all participants in a gown, without shoes, and after emptying their bladder/bowels. A stretch-resistant anthropometric tape (Gulick II model #67020, Gays Mills, WI, USA) with an indicator buckle to denote proper amount of tension applied to the tape was used for body circumference measurements. An average of two measures was recorded for each body site, unless the two measures differed by more than 1.0 cm, then a third measure was taken and the average of all three measures was used. Waist circumference was measured on a horizontal plane at the level of the iliac crest at the end of a normal expiration. Hip circumference was measured on a horizontal plane at the widest portion of the buttocks. Chest circumference was measured on a horizontal plan at the level of the nipples at the end of a normal expiration with the arms down at the subject’s sides. Upper arm (dominant arm) circumference was obtained at the measured midpoint between the shoulder and the elbow, with the arm hanging down the side of the body in a relaxed position. Thigh circumference was measured using the dominant leg at the widest portion of the thigh, which was typically the midpoint between the lower buttocks and the back of the knee.

Seated, resting blood pressure was obtained after the participant had been seated for at least 5 min. Participants refrained from smoking cigarettes (if applicable) or ingesting caffeine during the 30 min preceding the measurement. Blood pressure was measured using an automatic blood pressure device (Welch Allyn 300 Series, Skaneateles Falls, NY, USA).

### Body Composition

Total and regional fat mass and fat-free mass (lean mass and bone) were quantified by dual energy X-ray absorptiometry (DXA; GE Lunar Prodigy, enCORE software version 16, Madison, WI, USA) at baseline (week 0) and at weeks 4, 8, and 16. Total fat mass precision as reported by the manufacturer was <1.0%. A urine pregnancy test was performed on all women <60 years of age at screening and weeks 4, 8, and 16 prior to receiving a DXA scan.

### Laboratory Methods

Fasting blood samples were collected at screening for analysis of serum lipoprotein lipids, a comprehensive metabolic panel, and a complete blood count with automated differential performed by Elmhurst Memorial Hospital (EMH) Reference Laboratory (Elmhurst, IL, USA) according to their standard validated procedures, including the Standardization Program of the Centers for Disease Control and Prevention and the National Heart, Lung and Blood Institute for lipid measurements (14). Lipoprotein lipid assessments (milligrams per deciliter) included total cholesterol (TC), low-density lipoprotein cholesterol (LDL-C), high-density lipoprotein cholesterol (HDL-C), non-HDL-C (calculated as TC minus HDL-C), triglycerides, and the TC/HDL-C ratio. The LDL-C concentration in milligrams per deciliter was calculated according to the Friedewald equation as: LDL-C = TC − HDL-C − TG/5 (15).

### Quality of Life Assessments

Questionnaires designed to assess aspects of quality of life and sleep were administered at baseline and at weeks 4, 8, and 16. The Impact of Weight on Quality of Life-Lite questionnaire is a validated 31-item self-report measure used to assess obesity-specific quality of life domains, including physical function, self-esteem, sexual life, public distress, and work “over the past week” (16). Responses to questions within each domain were rated on a 5-point scale as “always true” (scored as 5), “usually true” (scored as 4), “sometimes true” (scored as 3), “rarely true” (scored as 2), or “never true” (scored as 1). Scores for each domain were obtained by summing responses to each individual question within that domain, and a total score was obtained by summing the domain scores. Higher scores indicate poorer quality of life.

The SF-36 health survey (17, 18) was used to assess more general aspects of quality of life, including physical functioning, role limitations caused by physical health problems, role limitations caused by emotional problems, social functioning, emotional well-being, energy/fatigue, pain, and general health perceptions “over the previous 4 weeks.” A higher score defines a more favorable health state.

The Leeds Sleep Evaluation Questionnaire (LSEQ), a validated 10-item self-report measure, was used to assess changes in sleep quality “compared to usual” over the course of the intervention pertaining to four aspects of sleep: Getting to Sleep, Quality of Sleep, Awakening from Sleep, and Behavior following Wakefulness (19). Responses to each question were measured on a visual analog scale using a 10-cm line with two extreme states defined at the ends of the line (e.g., “more difficult than usual”/“easier than usual”). Scores were averaged to provide a single score for each domain. A higher score indicates more favorable sleep outcomes.

### Statistical Analyses

A written statistical analysis plan was developed prior to the last subject completing the study before any data were analyzed. Statistical analyses were performed with SAS (SAS Institute, Cary, NC, USA, version 9.3). The current data was part of a four-arm parallel trial where three different commercial weight loss programs were each compared to the self-directed DASH diet control (20). Therefore, the original sample size calculation assumed a nominal α = 0.017 (one-sided) to account for up to three primary comparisons (each commercial weight loss program vs. control). A sample size of at least 35 per group was determined to provide 80% power to detect an effect size (*d*) of 0.72 for change in body weight between the groups. Additional participants were randomized to account for possible attrition. Participants were stratified across groups by age (18–35; 36–54; 55–70 years) and BMI (25.00–29.99; 30.00–44.99 kg/m^2^), and the total study sample included 70–80% women representing the typical profile of the commercial weight loss program consumer.

An intent-to-treat (ITT) analysis with last observation carried forward (LOCF) was used. Data at baseline are reported as mean ± SD and change from baseline data are reported as mean (95% confidence interval). Repeated measures analysis of covariance was used to assess differences between groups in the primary outcome variable (body weight) and continuous secondary outcome variables (body composition, body circumference parameters, and questionnaire data) at each post-randomization visit. Initial models contained terms for intervention, sex, stratification factors (BMI and age categories), and phase of menstrual cycle at start of intervention (follicular, luteal, or N/A), with baseline measures as covariates. Models were reduced using a backward selection method until only significant terms and/or intervention and baseline measure remained in the model (described for each parameter in footnotes of the results tables). Normality of residuals was investigated for each continuous outcome variable and if the normality assumption was rejected at the 1% level with the Shapiro–Wilk test, rank transformation was performed. Within each group, the paired *t*-test was used to determine if changes from baseline to each post-randomization visit in outcome variables were statistically significant. Safety assessments included an evaluation of post-randomization intervention-emergent adverse events.

## Results

### Participants

The disposition of participants throughout the study is shown in Supplementary Material. A total of 78 participants were randomized to the DASH diet (*n* = 40) or the commercial weight loss program (*n* = 38). Two participants randomized to the DASH diet discontinued the study at the baseline visit prior to beginning the assigned dietary intervention on the following day, so no LOCF imputation was applied for these individuals. Participants included predominantly Caucasian (70.6%) women (*n* = 61) and men (*n* = 17) with overweight and obesity, with a mean baseline age of 50.4 years, body weight of 95.7 kg, and BMI of 34.4 kg/m^2^. Additional demographic characteristics are presented in Table 1.

**Table 1:** Demographic characteristics at baseline of participants in a randomized trial allocated to a commercial weight loss program or a self-directed Dietary Approaches to Stop Hypertension diet.

Characteristic	Self-directed diet (*n* = 40)	Commercial program (*n* = 38)
	***n* (%)**	

Sex		
Male	9 (22.5)	8 (21.1)
Female	31 (77.5)	30 (78.9)
Race		
White	27 (67.5)	28 (73.7)
Black/African-American	9 (22.5)	7 (18.4)
Multiracial	3 (7.5)	0 (0.0)
Others	1 (2.5)	3 (7.9)

	**Mean ± SD**	

Age (years)	49.2 ± 11.6	51.5 ± 10.9
Body mass index (kg/m^2^)	35.2 ± 5.0	33.5 ± 4.5
Weight (kg)	99.2 ± 18.4	92.1 ± 16.0
Fat mass (kg)	44.4 ± 10.7	39.7 ± 9.1
Fat-free mass (kg)	55.0 ± 12.7	52.8 ± 10.5
Body fat (%)	45.9 ± 6.9	44.0 ± 6.2

### Body Weight

**Figure 1:**
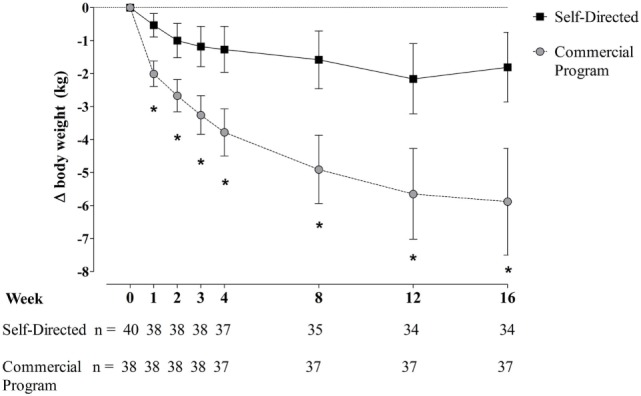
Change in body weight in men and women with overweight/obesity randomized to a commercial weight loss program or a self-directed Dietary Approaches to Stop Hypertension diet for a 16-week study period. Change from baseline data presented as mean ± 95% CI for the intent-to-treat sample population. Missing data were imputed using the method of last observation carried forward (LOCF). Two participants randomized to the self-directed diet discontinued the study at the baseline visit prior to beginning the assigned dietary intervention on the following day, so no LOCF imputation was applied for these individuals. Repeated measures analysis of covariance was used to assess differences between groups. Initial models contained terms for intervention, sex, stratification factors (body mass index and age categories), and phase of menstrual cycle at start of intervention (follicular, luteal, or N/A), with baseline measures as covariates. Models were reduced using a backward selection method until only significant terms and/or intervention and baseline measure remained in the model.

Changes in body weight at each time point over the 16-week study period are presented in Figure 1. Both groups lost a statistically significant amount of body weight from baseline to week 16, with the commercial weight loss program group losing more weight on average than the DASH group at every timepoint, about three times the weight loss of the DASH diet (*p* < 0.001 for all). Additionally, more participants in the commercial weight loss program group lost ≥5% of initial body weight by the end of the study (57.9% vs. 13.2%, *p* < 0.001).

### Body Composition

Weight loss with the commercial weight loss program was accompanied by larger reductions in total fat mass, and fat mass expressed as a percent of body weight (body fat%), compared to the DASH diet at every timepoint (Table 2). The commercial weight loss program group also experienced greater changes in regional body composition, including larger changes in android and abdominal visceral fat mass by week 16 (Table 2). Even though the DASH group lost less body weight, on average, than the commercial program group, approximately two-thirds of weight loss at week 16 was body fat.

**Table 2:** Changes in body weight and selected body composition parameters in men and women with overweight/obesity randomized to a commercial weight loss program or a self-directed Dietary Approaches to Stop Hypertension diet each for a 16-week study period.

Parameter	Self-directed diet (*n* = 38)^a^	Commercial program (*n* = 38)^a^
	
	Baseline = mean ± SD change (Δ) from baseline = mean (95% CI)	
**Body weight (kg)**		
Baseline	98.9 ± 18.6	92.1 ± 16.0
Δ Week 4	−1.3 (−2.0, −0.6)	−3.8 (−4.5, −3.1)*
Δ Week 8	−1.6 (−2.5, −0.7)	−4.9 (−5.9, −3.9)*
Δ Week 12	−2.2 (−3.2, −1.1)	−5.7 (−7.0, −4.3)*
Δ Week 16	−1.8 (−2.9, −0.8)	−5.9 (−7.5, −4.3)*
**Total fat mass (kg)**		
Baseline	44.4 ± 10.7	39.7 ± 9.1
Δ Week 4	−0.7 (−1.2, −0.1)	−1.9 (−2.4, −1.3)*
Δ Week 8	−1.0 (−1.7, −0.3)	−3.2 (−4.0, −2.4)*
Δ Week 16	−1.2 (−2.1, −0.4)	−4.4 (−5.7, −3.1)*
**Body fat (%)**		
Baseline	45.9 ± 6.9	44.0 ± 6.2
Δ Week 4	0.0 (−0.4, 0.4)	−0.3 (−0.8, −0.1)
Δ Week 8	−0.3 (−0.8, 0.2)	−1.2 (−1.8, −0.7)*
Δ Week 16	−0.4 (−1.0, 0.2)	−2.3 (−3.2, −1.4)*
**Android fat mass (kg)**		
Baseline	4.21 ± 1.17	3.83 ± 1.18
Δ Week 4	−0.10 (−0.28, 0.07)	−0.21 (−0.44, −0.03)*
Δ Week 8	−0.14 (−0.30, 0.03)	−0.49 (−0.69, −0.30)*
Δ Week 16	−0.24 (−0.47, −0.01)	−0.66 (−0.91, −0.40)*
**Abdominal visceral fat mass (kg)**		
Baseline	1.53 ± 0.64	1.43 ± 0.77
Δ Week 4^b^	−0.06 (−0.16, 0.04)	−0.10 (−0.23, 0.03)*
Δ Week 8	−0.01 (−0.13, 0.12)	−0.16 (−0.32, 0.00)*
Δ Week 16	−0.01 (−0.14, 0.13)	−0.21 (−0.40, −0.02)*

*^a^Sample size reflects the number of participants included in the intent-to-treat with last observation carried forward (LOCF) analysis. Two participants randomized to the self-directed diet discontinued the study at the baseline visit prior to beginning the assigned dietary intervention on the following day, so no LOCF imputation was applied for these individuals. Repeated measures analysis of covariance was used to assess differences between groups. Initial models contained terms for intervention, sex, stratification factors (body mass index and age categories), and phase of menstrual cycle at start of intervention (follicular, luteal, or N/A), with baseline measures as covariates. Models were reduced using a backward selection method until only significant terms and/or intervention and baseline measure remained in the model. The final models for body weight, total fat mass, body fat%, and abdominal visceral fat mass reduced to include terms for intervention and baseline measure as a covariate. For android fat mass, sex was also a significant term (*p* < 0.05) included the final model*.

*^b^n = 36 for abdominal visceral fat mass at week 4, as the visceral fat was not measured/quantified by DXA for two subjects in the self-directed DASH diet group*.

*p < 0.05, commercial program compared to self-directed diet.

### Body Circumference Measurements

Changes in body circumference parameters at each 4-week time point over the course of the study are presented in Table 3. While both groups experienced reductions in body circumference measurements, changes within the commercial weight loss program group were significantly greater (*p* < 0.05).

**Table 3:** Changes in body circumference parameters in men and women with overweight/obesity randomized to a commercial weight loss program or a self-directed Dietary Approaches to Stop Hypertension diet for a 16-week study period.

Parameter	Self-directed diet (*n* = 38)^a^	Commercial program (*n* = 38)^a^
	
	Baseline = mean ± SD change (Δ) from baseline = mean (95% CI)	
**Waist (cm)**		
Baseline	112.5 ± 12.7	108.4 ± 12.8
Δ Week 4	−1.5 (−2.6, −0.4)	−2.8 (−3.8, −1.8)*
Δ Week 8	−2.0 (−3.4, −0.5)	−4.0 (−5.2, −2.7)*
Δ Week 12	−2.1 (−3.5, −0.7)	−4.3 (−5.8, −2.9)*
Δ Week 16	−2.1 (−3.6, −0.6)	−4.1 (−5.8, −2.4)*^†^
**Hip (cm)**		
Baseline	121.6 ± 10.9	116.9 ± 10.4
Δ Week 4	−0.9 (−1.5, −0.3)	−2.3 (−2.9, −1.7)*
Δ Week 8	−1.1 (−1.9, −0.2)	−3.3 (−4.3, −2.4)*
Δ Week 12	−1.1 (−2.0, −0.3)	−3.9 (−5.0, −2.8)*
Δ Week 16	−1.3 (−2.3, −0.3)	−4.2 (−5.6, −2.9)*
**Chest (cm)**		
Baseline	115.9 ± 9.8	114.6 ± 10.3
Δ Week 4	−0.4 (−1.1, 0.2)	−3.0 (−3.8, −2.1)*
Δ Week 8	−0.7 (−1.4, 0.0)	−3.6 (−4.7, −2.6)*
Δ Week 12	−0.7 (−1.5, 0.1)	−3.9 (−5.1, −2.7)*
Δ Week 16	−0.2 (−1.0, 0.7)	−3.9 (−5.3, −2.6)*
**Upper arm (cm)**		
Baseline	37.6 ± 4.5	36.9 ± 4.3
Δ Week 4	−0.5 (−0.8, −0.2)	−1.2 (−1.4, −0.9)*
Δ Week 8	−0.7 (−1.1, −0.3)	−1.5 (−1.9, −1.1)*
Δ Week 12	−0.9 (−1.3, −0.4)	−1.7 (−2.2, −1.3)*
Δ Week 16	−0.9 (−1.4, −0.4)	−2.1 (−2.6, −1.6)*
**Thigh (cm)^b^**		
Baseline	62.7 ± 7.2	60.0 ± 5.6
Δ Week 4	−0.4 (−1.2, 0.4)	−1.5 (−2.1, −0.9)*
Δ Week 8	−0.9 (−1.7, −0.1)	−1.9 (−2.8, −1.0)*
Δ Week 12	−1.0 (−1.9, −0.2)	−2.2 (−3.1, −1.2)*
Δ Week 16	−1.4 (−2.4, −0.4)	−2.6 (−3.5, −1.6)*
**Total (cm)**		
Baseline	451.0 ± 37.5	436.9 ± 36.8
Δ Week 4	−3.7 (−5.6, −1.7)	−10.7 (−12.5, −9.0)*
Δ Week 8	−5.1 (−7.8, −2.5)	−14.3 (−17.3, −11.4)*
Δ Week 12	−5.6 (−8.3, −3.0)	−16.0 (−19.8, −12.3)*
Δ Week 16	−5.8 (−9.0, −2.6)	−16.9 (−21.5, −12.3)*

*^a^Sample size reflects the number of participants included in the intent-to-treat with last observation carried forward (LOCF) analysis. Two participants randomized to the self-directed diet discontinued the study at the baseline visit prior to beginning the assigned dietary intervention on the following day, so no LOCF imputation was applied for these individuals. Repeated measures analysis of covariance was used to assess differences between groups. Initial models contained terms for intervention, sex, stratification factors (body mass index and age categories), and phase of menstrual cycle at start of intervention (follicular, luteal, or N/A), with baseline measures as covariates. Models were reduced using a backward selection method until only significant terms and/or intervention and baseline measure remained in the model. The final model for all variables reduced to include only intervention and baseline measure as a covariate*.

*^b^One subject in the self-directed diet group was missing thigh circumference measurements at baseline, and therefore, the total body circumference parameter in this group was also missing a calculated baseline value*.

*p < 0.05, commercial program compared to self-directed diet.

*^†^p = 0.06, commercial program compared to self-directed diet*.

### Quality of Life and Sleep Quality

The total IWQOL score improved in both groups over the 16-week study period, but to a greater extent in the commercial weight loss program group compared to the DASH diet group at week 16 (Table 4) indicating improvements in weight associated quality of life in general for both groups. The individual domains generally followed the same pattern of improvement with weight loss in the commercial weight loss program group by the end of the study, with changes in physical function and self-esteem significantly different from the DASH diet.

**Table 4:** Responses to the Impact of Weight on Quality of Life-Lite questionnaire in men and women with overweight/obesity randomized to a commercial weight loss program or a self-directed Dietary Approaches to Stop Hypertension diet for a 16-week study period.

Questionnaire domain	Self-directed diet (*n* = 38)^a^	Commercial program (*n* = 38)^a^
	
	Baseline = mean ± SD change (Δ) from baseline = mean (95% CI)	
**Total IWQOL**		
Baseline	62.2 ± 19.0	64.1 ± 22.9
Δ Week 16	−7.0 (−11.3, −2.7)	−19.4 (−25.2, −13.7)*
**Physical function**		
Baseline	25.0 ± 8.3	25.2 ± 9.1
Δ Week 16	−3.5 (−5.1, −1.8)	−8.3 (−10.4, −6.1)*
**Self-esteem**		
Baseline	16.3 ± 6.1	18.1 ± 8.0
Δ Week 16	−1.5 (−3.3, 0.4)	−6.2 (−8.2, −4.3)*
**Sexual life**		
Baseline	6.9 ± 3.1	7.0 ± 3.6
Δ Week 16	−0.6 (−1.4, 0.2)	−1.7 (−2.6, −0.7)
**Public distress**		
Baseline	7.3 ± 3.2	6.9 ± 3.4
Δ Week 16	−0.8 (−1.6, 0.0)	−1.2 (−2.2, −0.2)
**Work**		
Baseline	6.7 ± 2.8	7.0 ± 3.2
Δ Week 16	−0.7 (−1.5, 0.1)	−2.1 (−3.1, −1.0)

*^a^Sample size reflects the number of participants included in the intent-to-treat with last observation carried forward (LOCF) analysis. Two participants randomized to the self-directed diet discontinued the study at the baseline visit prior to beginning the assigned dietary intervention on the following day, so no LOCF imputation was applied for these individuals. Repeated measures analysis of covariance was used to assess differences between groups. Initial models contained terms for intervention, sex, stratification factors (body mass index and age categories), and phase of menstrual cycle at start of intervention (follicular, luteal, or N/A), with baseline measures as covariates. Models were reduced using a backward selection method until only significant terms and/or intervention and baseline measure remained in the model. The final model for all variables reduced to include only intervention and baseline measure as a covariate*.

*p < 0.05, commercial program compared to self-directed diet.

Findings associated with the SF-36 questionnaire were, in general, variable both within and between groups, indicating the SF-36 might not have been a sensitive indicator of quality of life in this study sample (data not presented). Similarly, findings from the LSEQ were inconclusive as there were no significant differences between groups in sleep outcomes assessed.

### Blood Pressure

Both groups experienced reductions in systolic and diastolic blood pressure, as shown in Table 5, but there were no statistically significant differences in blood pressure changes between groups at any time point during the 16-week study period.

**Table 5:** Changes in blood pressure parameters in men and women with overweight/obesity randomized to a commercial weight loss program or a self-directed Dietary Approaches to Stop Hypertension diet for a 16-week study period.

Parameter	Self-directed diet (*n* = 38)^a^	Commercial program (*n* = 38)^a^
	
	Baseline = mean ± SD change (Δ) from baseline = mean (95% CI)	
**Systolic blood pressure (mm Hg)**		
Baseline	123.1 ± 12.7	123.0 ± 11.2
Δ Week 4	−7.3 (−11.2, −3.5)	−7.1 (−11.0, −3.1)
Δ Week 8	−5.8 (−9.2, −2.4)	−8.1 (−11.7, −4.6)
Δ Week 12	−4.1 (−8.1, −0.1)	−4.9 (−8.3, −1.4)
Δ Week 16	−5.3 (−9.0, −1.6)	−6.1 (−8.7, −3.4)
**Diastolic blood pressure (mm Hg)**		
Baseline	75.6 ± 9.6	77.0 ± 9.5
Δ Week 4	−3.7 (−6.3, −1.2)	−4.7 (−7.2, −2.1)
Δ Week 8	−3.8 (−6.8, −0.8)	−4.1 (−6.6, −1.7)
Δ Week 12	−1.9 (−4.5, 0.6)	−3.8 (−6.4, −1.2)
Δ Week 16	−2.8 (−5.2, −0.4)	−4.0 (−6.0, −2.0)

*^a^Sample size reflects the number of participants included in the intent-to-treat with last observation carried forward (LOCF) analysis. Two participants randomized to the self-directed diet discontinued the study at the baseline visit prior to beginning the assigned dietary intervention on the following day, so no LOCF imputation was applied for these individuals. Repeated measures analysis of covariance was used to assess differences between groups. Initial models contained terms for intervention, sex, stratification factors (body mass index and age categories), and phase of menstrual cycle at start of intervention (follicular, luteal or N/A), with baseline measures as covariates. Models were reduced using a backward selection method until only significant terms and/or intervention and baseline measure remained in the model. The final model for both variables reduced to include only intervention and baseline measure as a covariate*.

### Adverse Events

There were 15 adverse events reported in the self-directed DASH diet group, and 17 adverse events reported in the commercial program group; however, no adverse were reported as serious or severe, and no adverse events were deemed by the study physicians as related to either dietary intervention.

## Discussion

In this 16-week randomized parallel group study, adults with overweight and obesity who followed a comprehensive commercially available weight loss program experienced significantly greater reductions in body weight, body fat, and body circumference parameters compared to a self-directed diet modeled after the DASH dietary pattern with caloric restriction. These results suggest commercially available weight loss programs could be a reasonable option to consider, especially in a primary care setting where health-care practitioners may lack requisite nutrition training and/or time to engage with individuals seeking strategies for weight management.

The data from the present study are generally consistent with previous research showing that provision of portioned controlled foods as a dietary intervention for weight loss is an effective approach compared to conventional diets (5–8, 21–23). For example, a 2003 meta-analysis of six randomized controlled trials found that 3-month weight loss in participants consuming a partial meal replacement diet exceeded that of participants consuming a standard reduced calorie diet by 2.5–3.0 kg (~7% change from baseline) (5). These data, along with other studies, have been considered in the Academy of Nutrition and Dietetics (AND) Evidence Analysis Library (24) on research into Single Serving Portion Sized Meals (SSPSM) for weight management, which concluded that there is good evidence supporting the use of SSPSM for weight loss in adults. Furthermore, the 2009 AND position statement on weight management indicates strong evidence for the use of portion-controlled meal replacements for weight management (25).

In more recent studies of comparable design and duration to the present intervention, significant reductions in weight, body fat, and/or anthropometric parameters have been observed in participants following structured diets or commercially available programs incorporating portion-controlled foods compared to various control diets (22, 23). Participants following a commercially available program consisting of a reduced calorie diet achieved through the provision of portion-controlled foods, and delivered in a manner similar to the current study, lost more weight (−7.5 vs. −3.8 kg) than participants following a food-based energy-restricted diet at 26 weeks (22). These results are generally in-line with weight loss observed over a shorter time period (16 weeks) in the present study (−5.9 vs. −1.8 kg, commercial weight loss program vs. self-directed DASH diet). Another study published by Rock et al. (23) found that consumption of portion-controlled prepackaged lunch and dinner frozen entrees for 12 weeks, in conjunction with a prescribed reduced-energy diet and behavioral counseling, resulted in larger changes in weight compared to a standard self-selected diet in adults with overweight/obesity. However, it should also be mentioned that not all studies of similar duration and design have shown added benefits of portion-controlled foods relative to conventional energy-restricted diets on weight or related outcomes, but rather comparative effects or no statistically significant differences from conventional energy-restricted diets (26, 27).

It is noteworthy that the amount of weight loss observed in the present study was significantly different from the self-directed DASH diet by the first week and remained so throughout the course of the trial. However, it should be noted that the lower calorie intake level provided during the first week of the commercial program (1,000 kcal/d) was not the same as the 1,200 kcal/d (women) to 1,500 kcal/d (men) intake goal recommended during the first week of the self-directed diet. The lower calorie level during the first week of the commercial program was designed to promote an early larger initial weight loss to encourage compliance and continued weight loss with this program, and to understand how this compares to a more usual care approach to weight loss. This approach was deemed relevant, as large initial weight loss is meaningful from a clinical perspective, as previous research has shown that initial weight loss during the first few weeks of intervention is associated with better long-term weight loss outcomes (28–31). Additionally, analysis of pooled data from over 2,000 participants that took part in multiple phase 3 studies of a novel obesity pharmacotherapy demonstrated that participants who met or exceeded the threshold for clinically meaningful weight loss (≥5% initial body weight) at week 16 were more likely to maintain that amount of weight loss at 1 year (32). However, the present study did not examine longer term changes in body weight or composition, so additional research is needed to determine if the observed 16-week findings could contribute to long-term weight loss or maintenance of weight loss.

Other noteworthy findings from the present study include improvements in some self-reported weight associated quality of life parameters in response to the commercial weight loss program relative to the self-directed DASH diet. The inverse relationship between excess body weight or high BMI and poor health-related quality of life, and more specifically the prospective observation of improvements in specific aspects of quality of life with weight loss, has previously been shown (33, 34).

The present study had some limitations. It is recognized that there is no single best diet or program for all individuals seeking weight loss (35), and it should be acknowledged that the results of the present study were observed in predominantly Caucasian females with overweight and obesity. Therefore, the findings may not be generalizable to other specific segments of the population; however, the demographics of the study participants represent a population that reflects the average consumer of commercial programs to support weight loss, and therefore, the study results may have external validity. Additionally, in many dietary intervention studies, blinding is difficult or not possible and therefore participants and research staff were not fully blinded to intervention assignments. This is a potential limitation of the study design; however, the study biostatistician was blinded to randomization assignment prior to data analysis in an attempt to limit potential bias.

The choice of the control diet is also an important consideration when assessing strengths and limitations, and in this case, the choice to use the DASH diet deserves some discussion. While the DASH diet was originally designed to examine effects on blood pressure (12), investigators have noted weight loss in participants following the DASH diet with or without prescribed energy restriction in hypertension studies (36). Various forms of this diet have also been used in studies as a dietary intervention for weight loss (37). A systematic review and meta-analysis of 13 studies found that mean weight reduction with the DASH diet as the primary intervention was approximately 1.4 kg for interventions ranging 8–24 weeks in duration (37), which is similar to mean weight loss observed with the self-directed DASH diet in the present study by week 16 (−1.8 kg). Additionally, the DASH diet has been promoted in the popular press as an effective strategy for weight management (38, 39). Interestingly, although participants following the self-directed DASH diet experienced only modest weight loss on average, this group demonstrated statistically significant improvements in systolic and diastolic blood pressure, which is one indication the participants were adhering to the DASH dietary guidelines. Overall, the intent of using the DASH diet was to provide a well-studied, structured diet previously shown to promote weight loss that could be used to replicate usual care in a general clinic setting, wherein patients are typically provided with written materials or similar resources to support weight loss efforts with limited or no specific counseling or nutrition education provided by a health-care practitioner.

Additional limitations include no objective assessments of dietary intake or physical activity to potentially ascertain the independent or combined effects of these components on weight loss (or lack thereof), so results are attributable to the entirety of the commercial program (i.e., nutrition support and provision of foods). However, it should be noted that self-reported measures of dietary intake tend to underestimate true energy intakes, while self-reported measures of physical activity tend to overestimate true activity levels (references). Objectively measuring either variable is challenging to do with reasonable accuracy and precision in a cost-effective manner over a longer time frame in free-living individuals (references). That said physical activity levels could have been different among subjects within and between groups, which likely contributed to some of the variability in weight loss. Also, the study was conducted from April to October of the same calendar year, so potential seasonal differences (i.e., spring/fall vs. summer) in physical activity were not considered in our analyses and could be a limitation of the study. Lastly, there was a differential attrition rate between the two study groups, with the self-directed DASH diet having more drop-outs (*n* = 8; 15%) compared to the commercial program (*n* = 1; 3%). The higher rate of attrition in the self-directed DASH diet arm may suggest some individuals may not be able to follow a diet that does not provide pre-measured and prepared foods directly to participants; however, this is one reason for studying a self-directed diet vs. a commercial program, as the latter would be hypothesized to promote better compliance given the structured nature of the program with provision of foods.

One strength of the study was the use of DXA to quantify changes in body composition in addition to only examining changes in body weight. Another possible strength of the study was the low attrition rate, which resulted in limited missing data that may provide a more accurate representation of expected results when individuals fully complete a dietary program. The low attrition rate could have been due to several factors. Participants randomized to the DASH diet who completed the study were provided with a voucher to obtain 4 weeks of access to the commercial weight loss program, including foods, at no-cost to encourage study completion, which may have contributed to a lower attrition rate than observed in other weight loss studies. Of note, 4 weeks of access to the commercial program and provision of foods was the most the IRB would allow for these participants because the IRB deemed this a “bonus” form of compensation. The original intent was to provide the full 16 weeks to these participants. It is important to also consider that the typical consumer of commercial weight loss programs pays out of pocket and therefore may experience different outcomes compared to research participants who received all study foods and program support at no cost. The economics of weight management is a complicated issue and is beyond the scope of this study; however, it should be acknowledged that the potential impact of the cost of the program on weight outcomes in the present trial was not examined. The costs of a commercial weight loss program relative to those incurred with a self-directed advice based approach need to be carefully considered in the overall context of each individual’s unique weight management circumstances. Last, the structured dietary program, which consisted primarily of the provision of pre-portioned foods and shakes along with access to weight loss counselors (*via* phone at the discretion of participants) and other supporting print materials, may have supported overall compliance compared to that typically observed with usual care or conventional self-directed diets (40); however, compliance was not directly assessed in this study.

In summary, a commercial weight loss program resulted in significantly greater weight loss, fat loss, and reductions in body circumference parameters compared to a self-directed DASH diet over a 16-week intervention period. This program appears to be one approach available for health-care professionals to consider in working with a population with overweight/obesity, although the long-term effects on weight loss and weight loss maintenance are unknown. As such, future research is warranted in order to understand the effects of this program, and similar programs, on longer term changes in body weight, in particular weight loss maintenance, since many diets and weight management programs have variable success over periods of time greater than the 16-week period used in the present study.

## Ethics Statement

This study was conducted at Biofortis, Inc., Addison, IL, USA in accordance with the recommendations of Good Clinical Practice (GCP) Guidelines, the Declaration of Helsinki (2000), and the United States 21 Code of Federal Regulations. The protocol was approved by an accredited Institutional Review Board (IntegReview IRB; Austin, TX, USA). All participants provided signed informed consent and authorization for disclosure of protected health information before any study specific procedures were carried out.

## Author Contributions

CC contributed to the study design, development of the statistical analysis plan, analysis and interpretation of data, and writing the manuscript. CM and MK contributed to the study design, development of the statistical analysis plan, and critically reviewed the manuscript but were not involved in any data collection or analysis. VK was involved in the acquisition of data and in revising the manuscript critically for important intellectual content. All authors listed have contributed substantially to the work as noted and agreed to submit the manuscript.

## Conflict of Interest Statement

CM and MK are employees of Nutrisystem, Inc. CC and VK are employed by Biofortis, Inc., a contract research organization that conducted this study but did not directly receive funds from Nutrisystem.
