# Supplementary material for: A Commercially Available Portion-Controlled Diet Program Is More Effective for Weight Loss than a Self-Directed Diet: Results from a Randomized Clinical Trial
Source: Front Nutr. 2017 Nov 7;4:55. doi: 10.3389/fnut.2017.00055 (PMC5681920; doi:10.3389/fnut.2017.00055)
Supplement: Supplementary file 1 [file Data_Sheet_1.DOCX]

Supplementary Material

**A Commercially Available Portion Controlled Diet Program is more Effective for Weight Loss than a Self-Directed Diet:**

**Results from a Randomized Clinical Trial**

**Chad M. Cook, PhD^*1^, Courtney N. McCormick, MPH, RD^2^, Mandi** **Knowles, RD^2^, Valerie N. Kaden^1^**

^1^Biofortis, Inc., Addison, IL, USA

^2^Nutrisystem, Inc., Fort Washington, PA, USA

*** Correspondence:**

Chad M. Cook, PhD

E-mail: chad.cook@mxns.com

**1. Supplementary Data.**

Flow of subjects randomized to a commercial weight loss program or a self-directed Dietary Approaches to Stop Hypertension (DASH) diet each for a 16-week study period.

Screened (n = 243)

Did not meet entry criteria (n = 62)

Self-directed diet

(n = 40)

Commercial Program

(n = 38)

Intent-to-treat sample

(n = 38)^b^

Intent-to-treat sample

(n = 38)

Randomized (n = 181)

Week 1 (n = 38)

Week 2 (n = 38)

Week 3 (n = 38)

Week 4 (n = 37)

Week 8 (n = 35)

Week 12 (n = 34)

Week 16 (n = 34)

Discontinued (n = 6)

- Withdrew consent (n = 1)
- Lost to follow-up (n = 1)
- Other (n = 4)

Discontinued (n = 1)

- Withdrew consent (n = 1)

Week 1 (n = 38)

Week 2 (n = 38)

Week 3 (n = 38)

Week 4 (n = 38)

Week 8 (n = 37)

Week 12 (n = 37)

Week 16 (n = 37)

Data not presented (n = 103)^a^

- Excluded from analysis (n = 20)
- Subjects were randomized to one of two different programs not included in the present analysis (n = 83)

^a^ The data presented herein represent two arms from a randomized, four-arm parallel study that compared three different commercial weight loss programs each to a self-directed DASH diet control. Twenty (20) participants were originally randomized to weight loss plans with higher daily energy intake targets (1800 kcal for men and 1500 kcal/day for women) than those described in the present analysis. These weight loss plans were subsequently discontinued after the study began randomizing participants. As a result, intent-to-treat (ITT) with last observation carried forward (LOCF) analyses were completed on the participants randomized to weight loss plans with the same calorie levels for women and men (1200 kcal/day for women and 1500 kcal/day for men) in order to compare groups with the same daily energy intake targets.

^b^ Two participants randomized to the self-directed diet discontinued the study at the baseline visit prior to beginning the assigned dietary intervention the following day, so LOCF imputation was not applied as there were no post-baseline data available for imputation.
